# Evaluation of Light-Dependent Photosynthetic Reactions in *Reynoutria japonica* Houtt. Leaves Grown at Different Light Conditions

**DOI:** 10.3389/fpls.2021.612702

**Published:** 2021-08-04

**Authors:** Selma Mlinarić, Lidija Begović, Neven Tripić, Antonija Piškor, Vera Cesar

**Affiliations:** ^1^Department of Biology, Josip Juraj Strossmayer University of Osijek, Osijek, Croatia; ^2^Faculty of Dental Medicine and Health, Josip Juraj Strossmayer University of Osijek, Osijek, Croatia

**Keywords:** Japanese knotweed, invasive species, modulated 820 nm reflectance, JIP-test, total driving forces, non-photochemical quenching

## Abstract

The Japanese knotweed (*Reynoutria japonica* Houtt.) is considered as one of the most aggressive and highly successful invasive plants with a negative impact on invaded habitats. Its uncontrolled expansion became a significant threat to the native species throughout Europe. Due to its extensive rhizome system, rapid growth, and allelopathic activity, it usually forms monocultures that negatively affect the nearby vegetation. The efficient regulation of partitioning and utilization of energy in photosynthesis enables invasive plants to adapt rapidly a variety of environmental conditions. Therefore, we aimed to determine the influence of light conditions on photosynthetic reactions in the Japanese knotweed. Plants were grown under two different light regimes, namely, constant low light (CLL, 40 μmol/m^2^/s) and fluctuating light (FL, 0–1,250 μmol/m^2^/s). To evaluate the photosynthetic performance, the direct and modulated chlorophyll *a* fluorescence was measured. Plants grown at a CLL served as control. The photosynthetic measurements revealed better photosystem II (PSII) stability and functional oxygen-evolving center of plants grown in FL. They also exhibited more efficient conversion of excitation energy to electron transport and an efficient electron transport beyond the primary electron acceptor Q_A_, all the way to PSI. The enhanced photochemical activity of PSI suggested the formation of a successful adaptive mechanism by regulating the distribution of excitation energy between PSII and PSI to minimize photooxidative damage. A faster oxidation at the PSI side most probably resulted in the generation of the cyclic electron flow around PSI. Besides, the short-term exposure of FL-grown knotweeds to high light intensity increased the yield induced by downregulatory processes, suggesting that the generation of the cyclic electron flow protected PSI from photoinhibition.

## Introduction

In the natural environment, plants are exposed to fluctuations of quantity and quality of the incident light. They can adjust the physiological and biochemical processes to sudden changes in light conditions. However, in the experimental conditions, plants are often grown at the continuous light regime. At low-light conditions, they have to adjust the functioning to use the available light efficiently for the optimal photosynthesis, while at high-light conditions, they have to protect themselves from photoinhibition damage. Often, such adjustments include structural changes at different levels, including, thylakoid, photosystem (PS), pigment, and/or protein (Keren et al., 1997; Lichtenthaler et al., 2007; Kouřil et al., 2013). Contrary to the short-term adaptation to low–light conditions, the long-term strategies involved different structural changes, such as increased leaf mass and thickness, increased amount of thylakoids, or/and higher chlorophyll content (Lichtenthaler and Burkart, 1999; Lichtenthaler et al., 2007). Low light was shown to induce alterations in the photosynthetic apparatus in beech and barley, which resulted in the limitation of the electron transport due to the lower amount of electron carriers and due to a lower connectivity of PSII units in shaded leaves (Desotgiu et al., 2012; Živčak et al., 2014). However, the response to the fluctuating-light (FL) conditions depends on the environmental and experimental conditions, as well as on the species, developmental stage, and physiological factors of the plants (Yin and Johnson, 2000; Kaiser et al., 2018), and usually includes the reprogramming of gene expression connected to the photosynthetic processes (Armbruster et al., 2017; Schneider et al., 2019) and stomatal acclimation (Matthews et al., 2018; Yamori et al., 2020). When plants grow in natural, FL, they have to develop the long-term acclimation responses that differ from those found in plants growing at constant high-light or constant low-light (CLL) conditions (Schneider et al., 2019). Although, recently, the technology and availability of illumination systems are more acceptable and they could simulate natural light conditions, sudden changes in light intensity, clouds, or even wind in nature could occur in less than a second. Therefore, to understand how plants behave in such environments, it is desirable to study the photosynthetic processes under natural environmental conditions.

The Japanese knotweed (*Reynoutria japonica* Houtt.) is one of the most widespread invasive species in Croatia (Boršić et al., 2008; CABI, 2019; FCD, 2020). It is a fast-growing and perennial shrub, very invasive due to its rapid spread in various ecosystems, and very difficult to remove. It is characterized by the ability of the exceptional reproduction and the rapid physiological adaptation to the conditions in the new environment (Spiering, 2011). The Japanese knotweed can also be potentially beneficial to the human society. Its high resistance and efficient accumulation of heavy metals from the environment make it an ideal candidate for soil phytoremediation. It has proven to be an acceptable source of food for humans, domestic animals, and bees, and its metabolism creates compounds that are of potential importance for the herbicide medicine and industry (Beerling et al., 1994; Barney et al., 2006).

One of the most important mechanisms that allows invasive plants to achieve success in a variety of environmental conditions is attributed to the higher photosynthetic rate compared with the native plants (Li and Xiao, 2012; Bajwa et al., 2016). An important aspect of the monitoring and detection of plant responses and their survival under natural conditions is the estimation of their physiological status. Recently, the chlorophyll *a* fluorescence has been extensively used as a non-invasive, very sensitive, and fast method for the estimation of the photosynthetic performance that can provide a reliable source of information on plant conditions (Goltsev et al., 2016; Bussotti and Pollastrini, 2017; Mlinarić et al., 2017; Pollastrini et al., 2017; Kalaji et al., 2018b; Begović et al., 2020).

Since the Japanese knotweed is a heliophilic species, it is adapted to grow under the conditions of increased light intensity. Due to its fast-spreading nature and by creating monocultures, it has become a serious threat to the biodiversity. It is easily cultivated, can grow in various types of soils, and can adapt to a large scale of environmental factors (Beerling et al., 1994; Barney et al., 2006). As an invasive species, it is capable of developing certain adaptations to less favorable conditions. However, one of the major environmental factors that can control its performance is the availability of light. It affects the above- and below-ground biomass of knotweed directly by reducing its performance and, consequently, its invasiveness (Dommanget et al., 2013; Dommanget et al., 2019). Specifically, the below-ground system of the knotweeds presents the majority of its biomass. Plants grown in high-light conditions, in comparison with those grown in low-light conditions, allow the allocation of more resources to the below-ground system, indicating a strong effect of light, hence, enabling colonization and competitiveness of the Japanese knotweed (Price et al., 2002). The most recent study revealed that the Japanese knotweed adopts differential strategies of growth and space occupancy when grown in full sunlight and in shaded habitats (Martin et al., 2020). Therefore, we hypothesized that plants grown at different light regimes, e.g., CLL and fluctuating natural light, would develop certain adaptations to such conditions in photosynthetic reactions. An efficient photosynthesis was recognized to be one of the most important mechanisms that allow invasive plants to achieve success in various environmental conditions (Bajwa et al., 2016). Thus, the main objective of this study was to determine the influence of different illumination regimes on the efficiency of the photosynthetic apparatus and to gain detailed insight into its functioning in the invasive Japanese knotweed by using mainly non-destructive methods, simultaneous measurements of prompt fluorescence, modulated 820 nm reflection (MR), and saturating pulse method, as well as by the determination of the content of the photosynthetic pigment. To our knowledge, the obtained results in this investigation will reveal the most detailed insight into the light-driven reactions in the invasive Japanese knotweed and the adaptations of the photosynthetic apparatus to CLL and FL conditions. Therefore, our investigation results could contribute to a better understanding of mechanisms that play a role in the success of this invasive species.

## Materials and Methods

### Experimental Setup

Rhizomes of the Japanese knotweed (*R. japonica* Houtt.) were planted in a mixture of commercial soil and sand (3:1) in six plastic containers (50 cm × 19 cm × 16.5 cm). The soil used was natural peat (pH = 5.5–7) with the addition of the fertilizer (Supplementary Table 2). Three of them were placed in the room near the window facing south, exposed directly to sunlight. The light intensity (Quantitherm QRT1 light meter, Hansatech, United Kingdom) varied from 30 to 1,250 μmol/m^2^/s. The photoperiod changed from 11 to 16 h of FL, while the temperature was 23°C ± 1°C. A detailed information on the distribution of the light throughout the day, the changes in photoperiod, light intensity, and zenith angle that occurred for 110 days is shown in Supplementary Table 1. The other three containers were placed in the growth chamber with the day/night photoperiod of 16/8 h (day/night), CLL intensity (i.e., 40 μmol/m^2^/s), and a constant temperature of 23°C ± 1°C. The combination of warm white light (i.e., 3,000 K), cool white light (i.e., 4,000 K), and cool daylight (i.e., 6,500 K) from fluorescent tubes (Osram, Munich, Germany) provided a range of visible-light spectra within the visible range between 300 and 700 nm, with maximum peaks at blue, green, and red parts of the spectra (Supplementary Figure 1). The plants grown in CLL were used as the control group. They were watered regularly. They started to emerge at 2 weeks after the planting. In each container, at least five plants were growing from one rhizome. The measurements were carried out 110 days after planting on the fully grown leaves (third leaf from the top of the plant).

### Simultaneous Measurements of the Prompt Fluorescence and Modulated 820 nm Reflection

The prompt chlorophyll *a* fluorescence (PF) and MR were simultaneously recorded *in vivo* on five plants in each container (*n* = 15) using Multichannel Plant Efficiency Analyser, M-PEA (Hansatech Instruments, Norfolk, United Kingdom). All measurements were performed on attached, fully dark-adapted leaves (for 30 min). During the measurements, the leaves were exposed to a pulse of high intensity red light-emitting diode (LED) at 625 nm and intensity of up to 5,000 μmol photons/m^2^/s to ensure an effective light saturation of exposed leaf surface (i.e., 4-mm diameter). Recorded PF data were analyzed using the JIP-test that represents the translation of the original data to biophysical parameters that quantify the energy fluxes through PSII (Strasser et al., 2000; Strasser et al., 2004). The OJIP transients are presented as mean values of 15 measurements for each group of plants. To evaluate the condition of the photosynthetic apparatus in CLL- and FL-grown Japanese knotweed plants, the selected structural and functional parameters calculated from the JIP-test were chosen. The description of the calculated OJIP test parameters is given in Table 1. To compare the recorded OJIP transients for specific events in the OP, OK, OJ, JP, and IP phases, the difference in the relative variable fluorescence (ΔVt) was calculated and presented as a difference ΔV_OP_, ΔV_OK_, ΔV_OJ_, ΔV_JP_, and ΔV_IP_ normalized to the control (CLL-grown plants) (Yusuf et al., 2010; Da̧browski et al., 2019). The total driving force (DF_total_) of the total photosynthetic electron transport, shown as log PI_total_, was summed up by the corresponding partial DFs: log γ_RC_/(1-γ_RC_), log φ_P__0_/(1-φ_P__0_), log ψ_E__0_/(1-ψ_E__0_), and log δ_R__0_/(1-δ_R__0_) (van Heerden et al., 2007). The MR measurements for high-quality P700 reflectance were performed by using modulated 820-nm LED. From the MR signal of the reflected beam, the MR/MR_0_ ratio was calculated. The first reliable MR measurement (MR_0_) value was taken at 0.7 ms (Strasser et al., 2010; Oukarroum et al., 2013; Salvatori et al., 2014; Salvatori et al., 2015). The parameters and formulas used are listed in Table 1.

**TABLE 1:** Description of used JIP-test parameters.

Prompt fluorescence (PF)	
**Technical parameters**	
F_0_	Fluorescence intensity at 20 μs
F_m_	Maximal fluorescence intensity
F_t_	Fluorescence intensity at time *t* after the onset of actinic illumination
F_v_	Maximal variable fluorescence
t_Fm_	Time to reach maximal fluorescence intensity F_m_
Area	Total complementary area between the fluorescence induction curve and F = F_m_
S_m_	Normalized total area above OJIP curve, reflecting multiple-turnover events
S_m_/t_Fm_	Index quantifying the average excitation energy of open RCs from *t* = 0 to t_Fm_
N	Turnover number
M_0_	Initial slope of the curve at the origin of the relative variable fluorescence rise
Vt	Relative variable fluorescence at time *t*
**Density and overall grouping probability of RCs**	
RC/CS_0_	Measure for Q_A_^–^ reducing RCs per excited leaf cross-section (CS)
Q_B_ reducing centers	The fraction of Q_B_ reducing reaction centers
Non-Q_B_ reducing centers	The fraction of non-Q_B_ reducing reaction centers
OEC centers	The fraction of Oxygen Evolving Complexes (OEC)
P_2__G_	Overall grouping probability for the use of the absorbed energy in photochemical reactions
**Quantum efficiencies and flux ratios**	
φ_P__0_ = TR_0_/ABS	Maximum quantum yield of primary photochemistry, the probability that an absorbed photon will be trapped by the PSII RC and will reduce one Q_A_
ψ_E__0_ = ET_0_/TR_0_	Electron transport efficiency, the probability that an absorbed photon will enter the electron transport chain
φ_E__0_ = ET_0_/ABS	Probability that a photon trapped by the PSII RC enters the electron transport chain
δ_R__0_ = RE_0_-ET_0_	Probability that an electron is transported from reduced PQ to the electron acceptor side of PSI
φ_R__0_ = RE_0_/ABS	Quantum yield of electron transport from Q_A_^–^ to the PSI end electron acceptors
ABS/RC	Effective antenna size of an active reaction center (RC). Expresses the total number of photons absorbed by Chl molecules of all RC divided by the total number of active RCs
ET_0_/RC	Electron transport in an active RC
TR_0_/RC	Maximal trapping rate of PSII. Describes the maximal rate by which excitation is trapped by the RC
DI_0_/RC	Effective dissipation in an active RC
RE_0_/RC	Electron flux reducing end electron acceptors at the PSI acceptor side per RC
**Performance indices and driving forces**	
PI_ABS_ = γ_RC_/(1-γ_RC_) x φ_P__0_/(1-φ_P__0_) x ψ_E__0_/(1-ψ_E__0_)	Performance index (potential) for energy conservation from photons absorbed by PSII to the reduction of intersystem electron acceptors.
PI_total_ = γ_RC_/(1-γ_RC_) x φ_P__0_/(1-φ_P__0_) x ψ_E__0_/(1-ψ_E__0_) x δ_R__0_/(1-δ_R__0_)	Performance index (potential) for energy conservation from photons absorbed by PSII to the reduction of PSI end acceptors
DF_total_ = log PI_total_	Total driving forces for photosynthesis of the observed system, created by summing up the partial driving forces for each of the several bifurcations
**Modulated reflection (MR)**	
V_ox_	Rate of P700 and PC oxidation, calculated as the maximum slope decrease of MR_t_/MR_0_
V_red_	Rate of P700 and PC re-reduction, calculated as the maximum slope increase of MRt/MR_0_
MR_min_	A transitory steady state, with equal oxidation and re-reduction rates of P700 and PC, calculated as the minimum of MRt/MR_0_

### Double-Pulse Method

For the calculation of Q_B_-reducing and non-Q_B_-reducing centers, the double-hit measurement protocol was used. The protocol was set up at M-PEA, and it was measured simultaneously with PF and MF. After the first pulse that was used to measure PF followed a second pulse after the dark period of 500 ms. The relative fraction of Q_B_-reducing and non-Q_B_-reducing centers was calculated as described in the studies of Mathur et al. (2011) and Tomar et al. (2015).

### Saturation Pulse Method

The effect of light intensity on the PSII activity was determined by measuring chlorophyll *a* fluorescence *in vivo* on two randomly selected leaves per container using amplitude-modulated fluorometer MiniPAM (Walz, Effeltrich, Germany). The minimal (F_0_) and maximal (F_m_) fluorescence yields were measured in the dark-adapted leaves (30 min). Same parameters (F′) and (F_m_′) were measured at the photosynthetically active photon flux density (PPFD) at 100, 250, 500, 1,000, and 2,000 μmol photons/m^2^/s. The following parameters were calculated: maximum quantum yield of PSII, effective quantum yield of PSII [Y(PSII)], the relative rate of the electron transport (relETR; Genty et al., 1989), non-photochemical quenching (NPQ; Bilger and Björkman, 1990), quantum yield induced by downregulatory processes in PSII [Y(NPQ)], and quantum yield of non-regulated energy dissipated in PSII [Y(NO)] (Kramer et al., 2004).

### Determination of the Photosynthetic Pigments

After the measurements of PF and MR, the same leaves were used for the determination of the concentration of photosynthetic pigments. The leaves were powdered using liquid nitrogen, and the photosynthetic pigments were extracted using cold acetone. The concentrations of chlorophylls (Chl *a* and Chl *b*) and carotenoids (Car) were determined spectrophotometrically (Specord 40, Analytik Jena, Germany) at 470, 661.6, and 644.8 nm. The total chlorophyll (Chl *a* + *b*) concentration, as well as the chlorophyll *a* and *b* ratio (Chl *a*/*b*) and the Chl *a* + *b* to Car ratio (Chl *a* + *b*/Car), was calculated (Lichtenthaler, 1987).

### Data Analysis

The Student’s *t*-test was used to analyze the statistical differences between the leaves exposed to CLL and FL conditions. The asterisk (^∗^) indicates a significant difference between the compared parameters. For simultaneous DF and MR, as well as for double-pulse measurements, five leaves per container were used (*n* = 15). For the photosynthetic pigment concentration, same leaves were collected into composite sample and six (*n* = 6) replicates were measured per treatment. For modulated fluorescence measurements, two leaves per container were measured (*n* = 6). The difference between the parameters measured in CLL and FL plants, as well as between the parameters at different PPFD, was analyzed by one-way analysis of variance (ANOVA), followed by the Fisher’s least significant difference (LSD) *post hoc* test. The results were expressed as means ± standard deviation (SD), and the differences were considered significant at *p* < 0.05. For all statistical analyses, Statistica 13.4.0.14 software (TIBCO Software Inc., Palo Alto, CA, USA) was used.

## Results

### Analysis of Prompt Chlorophyll *a* Fluorescence Transients and Parameters of the JIP-Test

**FIGURE 1:**
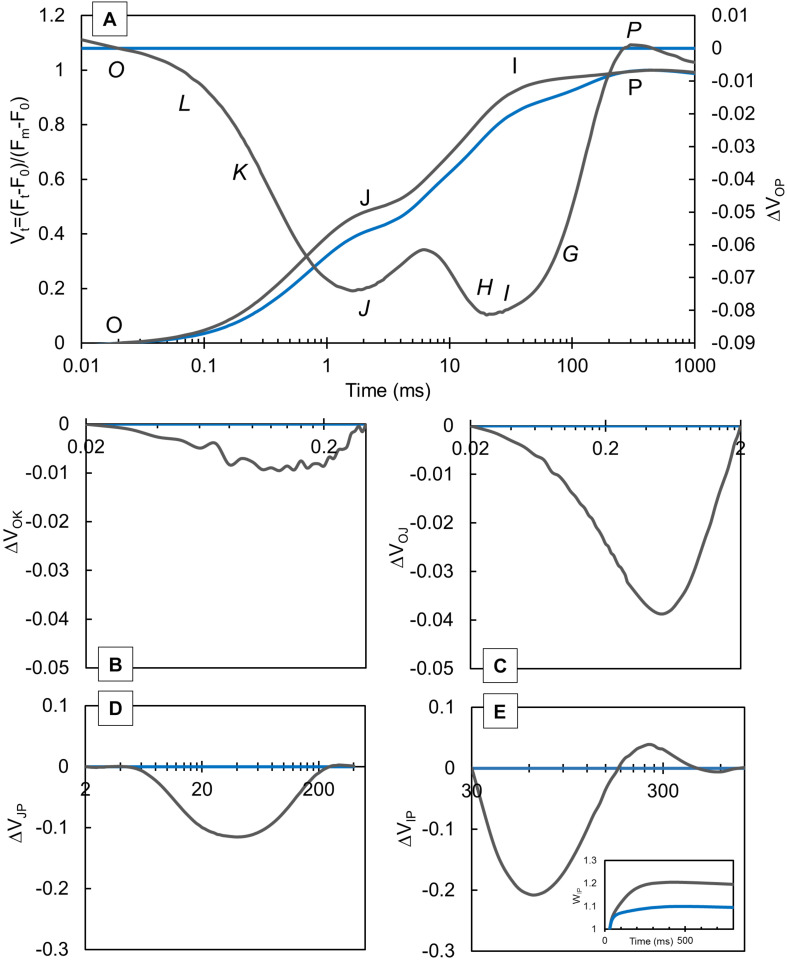
Variations in the shape of the transient curves of the chlorophyll *a* fluorescence measured in the Japanese knotweed (*Reynoutria japonica* Houtt.) leaves exposed to constant low light (blue lines) and fluctuating light (gray lines). Each curve represents the average kinetics of 15 measurements (*n* = 15) per treatment. Average fluorescence data were normalized between OP **(A)**, OK **(B)**, OJ **(C)** JP **(D)**, and IP **(E)** steps and plotted as difference kinetics ΔVt in a different time range. Average values measured in constant low light were used as referent values. The relative variable fluorescence transient, V_t_ **(A)**, shows typical O-J-I-P steps, while in difference kinetics, ΔV_OP_, specific bands *O-L-K-J-I-H-G-P* can be distinguished.

Prompt chlorophyll *a* fluorescence and MR were measured in knotweed plants grown at CLL and FL conditions. The curve normalized between O and P steps (Figure 1A) showed higher values in FL-grown plants compared with CLL–grown plants. The ΔVt (Figures 1A–E) was calculated as the difference between FL-grown and CLL–grown plants, which was used as a reference for data normalization. Our results revealed negative ΔL (Figure 1B), ΔK (Figure 1C), and ΔH (Figure 1D) bands, while the ΔG band (Figure 1E) showed a negative amplitude followed by a slight positive inflection in FL-grown knotweeds. The V_IP_ ≥ 1, plotted in the 30–300 ms range (Figure 1E, insert), showed a higher amplitude in FL-grown plants.

The fluorescence intensity at 20 s (F_0_) (Table 2) showed significantly lower values in FL-grown plants compared with CLL-grown plants, while the F_m_ showed no difference between the two plant groups. The total complementary area between the curves of fluorescence induction and F_m_ (Area) revealed almost two times higher values in FL-grown plants compared with CLL-grown plants. The time to reach the maximal fluorescence intensity (t_Fm_) as well as the M_0_, the initial slope of relative variable fluorescence, was low in FL-grown plants when compared with CLL-grown plants. The parameters including S_m_, that provides an amount of the energy that is needed to close all reaction centers, S_m_/t_Fm_, that describes the average fraction of open reaction centers during the time needed to complete their closure, N, the turnover number, and the fraction of oxygen-evolving complex (OEC) revealed significantly higher values in FL-grown plants than in CLL–grown plants. The density of active reaction centers (RC) per cross-section, RC/CS_0_, the fraction of Q_B_ and non-Q_B_ reducing centers and an overall grouping probability, P_2__G_, showed no significant difference between two differentially grown plant groups. However, the variable fluorescence measured at all chosen time points, V_L_, V_K_, V_I_, and V_J_, showed significantly lower values in FL-grown plants compared with CLL–grown plants.

**TABLE 2:** Selected parameters of the chlorophyll *a* fluorescence, characterizing PSII functioning gained from measurements of the Japanese knotweed (*Reynoutria japonica* Houtt.) leaves exposed to constant low and fluctuating light.

	Constant low light	Fluctuating light	*t*-value	*p*
F_0_	6540.467 ± 1305.285	5347.333 ± 581.853	–3.233	0.003*
F_m_	26239.000 ± 2584.880	25021.933 ± 2636.281	–1.277	0.212
F_v_/F_0_	3.095 ± 0.474	3.687 ± 0.225	4.371	< 0.001*
F_0_/F_m_	0.247 ± 0.029	0.214 ± 0.011	–4.208	< 0.001*
Area	279006.792 ± 32506.227	464173.496 ± 40850.223	13.737	< 0.001*
t_Fm_	500.000 ± 65.465	432.667 ± 73.724	–2.644	0.013*
S_m_	14.238 ± 1.948	23.771 ± 2.515	11.606	< 0.001*
S_m_/t_Fm_	0.029 ± 0.004	0.056 ± 0.011	9.365	< 0.001*
N	4.448 ± 0.416	6.574 ± 0.605	8.014	< 0.001*
M_0_	0.611 ± 0.109	0.470 ± 0.041	–4.708	< 0.001*
RC/CS_0_	3845.759 ± 450.251	3626.533 ± 268.331	1.620	0.116
OEC fraction	0.653 ± 0.040	0.683 ± 0.030	2.308	0.029*
Q_B_ reducing RCs	0.600 ± 0.035	0.606 ± 0.023	0.492	0.627
non-Q_B_ reducing RCs	0.400 ± 0.035	0.394 ± 0.023	–0.492	0.627
P_2__G_	0.284 ± 0.121	0.304 ± 0.091	0.501	0.143
V_L_	0.079 ± 0.019	0.059 ± 0.005	–3.876	< 0.001*
V_K_	0.167 ± 0.031	0.129 ± 0.011	–4.585	< 0.001*
V_J_	0.478 ± 0.038	0.408 ± 0.042	–4.776	< 0.001*
V_I_	0.909 ± 0.017	0.827 ± 0.033	–8.592	< 0.001*

*Data are presented as mean ± SD.*

An asterisk (*) represents a significant difference at *p* ≤ 0.05 (using the Student’s *t*-test). For parameter abbreviations, see Table 1.

**FIGURE 2:**
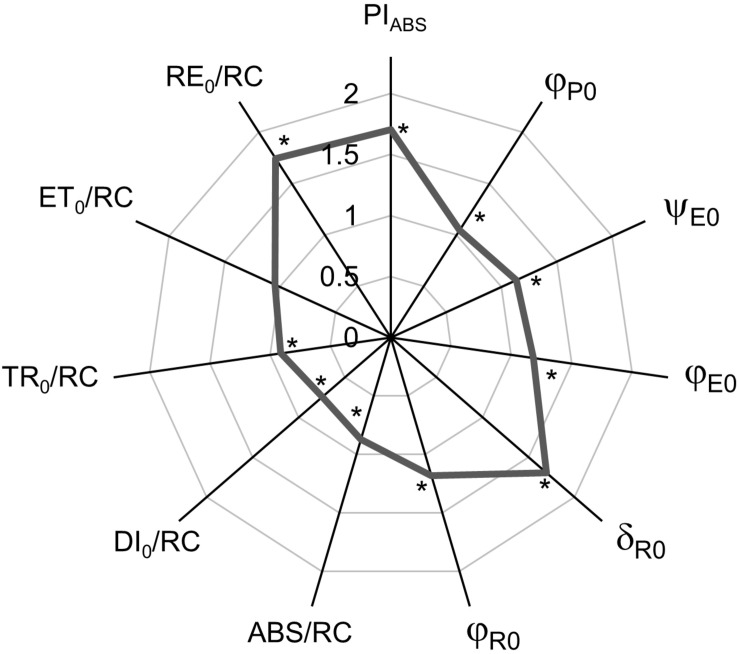
Spider plots display the normalized values of selected parameters of chlorophyll *a* fluorescence characterizing PSII functioning: performance index (PI_ABS_), quantum yields (φ_P__0_, ψ_E__0_, φ_E__0_, δ_R__0_, and φ_R__0_), and specific energy fluxes per Q_A_^–^ reducing PSII RC (ABS/RC, DI_0_/RC, TR_0_/RC, ET_0_/RC, and RE_0_/RC) of Japanese knotweed (*Reynoutria japonica* Houtt.) leaves exposed to fluctuating light (gray line). The values for plants grown in fluctuating light were shown as the difference compared with low-light-grown plants (control = 1). The curve represents the mean values of 15 replicates. The asterisk (*) represents a significant difference at *p* ≤ 0.05 (using the Student’s *t*-test) compared with control.

The spider plot (Figure 2) represents the normalized curves of the calculated biophysical parameters derived from the JIP-test which characterize the functioning of PSII. Results are represented as the difference between FL-grown plants and CLL-grown plants that were used as control. The performance index (PI_ABS_) showed significantly higher values of FL-grown plants compared with CLL-grown plants. The quantum yields and probabilities (φ_P__0_, ψ_E__0_, φ_E__0_, δ_R__0_, and φ_R__0_) were significantly higher in FL-grown plants when compared with CLL-grown plants. The specific energy fluxes per reducing PSII RCs, absorption (ABS/RC), and dissipation (DI_0_/RC) were significantly lower in FL-grown plants; trapping (TR_0_/RC) and electron flux reducing end electron acceptors at the PSI acceptor side (RE_0_/RC) showed significantly higher values, while the electron transport further than Q_A_^–^ (ET_0_/RC) showed no significant difference compared with CLL-grown plants.

### Total Driving Forces

**FIGURE 3:**
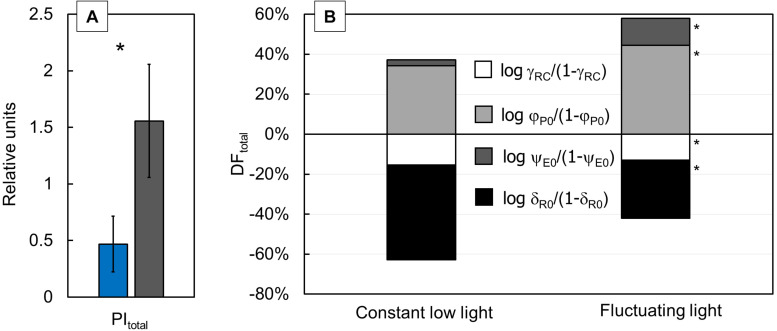
Difference of the performance index for energy conservation from exciton to the reduction of the PSI end acceptors [PI_total_ **(A)**] was measured in the Japanese knotweed (*Reynoutria japonica* Houtt.) leaves exposed to constant low light (blue bar) and fluctuating light (gray bar). Variation in the total driving forces [DF_total_ **(B)**] for each group of plants was calculated by summing up their partial driving forces: log γ_RC_/(1–γ_RC_) (white), log φ_P__0_/(1–φ_P__0_) (light gray), log ψ_E__0_/(1–ψ_E__0_) (dark gray), and log δ_R__0_/(1-δ_R__0_) (black). Represented values are the mean of 15 replicates per treatment. The asterisk (*) represents a significant difference at *p* ≤ 0.05 (using the Student’s *t*-test); error bars represent ± SD.

The performance index for the energy conservation from the exciton to the reduction of PSI end acceptors (PI_total_) showed three times higher values in FL-grown plants than in CLL-grown plants (Figure 3A). DF_total_ (Figure 3B) for photosynthesis in the observed system are presented as corresponding partial DFs: log γ_RC_/(1-γ_RC_), log φ_P__0_/(1-φ_P__0_), log ψ_E__0_/(1-ψ_E__0_), and log δ_R__0_/(1-δ_R__0_). All calculated partial DFs showed a significant difference between FL-grown and CLL-grown plants. The PI_total_ (Figure 3A) in FL–grown plants increased due to an increase in log γ_RC_/(1-γ_RC_) and log ψ_E__0_/(1-ψ_E__0_), as well as less negative values of log δ_R__0_/(1-δ_R__0_).

### Analysis of Modulated 820 nm Reflection Transients

**FIGURE 4:**
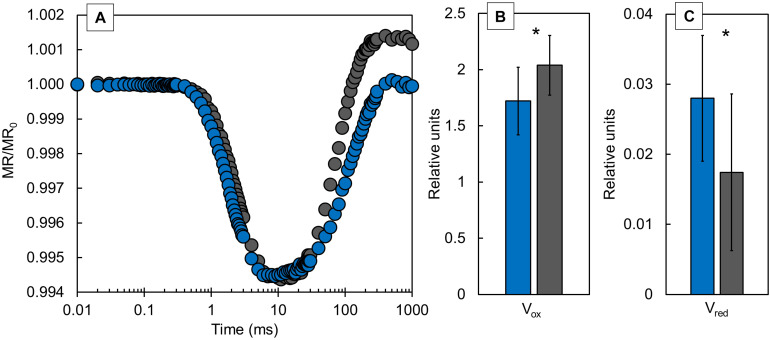
The kinetics of modulated 820 nm reflection (MR) normalized to MR_0_ **(A)**, values of the oxidation rate of PC and P700 [V_ox_ **(B)**], and re-reduction rate of PC^+^ and P700^+^ [V_red_ **(C)**] were measured in the Japanese knotweed (*Reynoutria japonica* Houtt.) leaves exposed to constant low light (blue) and fluctuating light (gray). The represented values are the mean of 15 replicates per treatment. The asterisk (*) represents a significant difference at *p* ≤ 0.05 (using the Student’s *t*-test); error bars represent ± SD.

Modulated 820 nm reflection signals (Figure 4A) were presented as MR/MR_0_ ratio. The differences in kinetics at 820 nm reveal the redox states of P700 and PC. The typical MR transient comprises of fast decreasing phase from MR_0_ to MR_min_ (at ∼0.7–7 ms, respectively) and a slow increasing phase from MR_min_ to MR_max_ (at ∼300 ms). Our results showed a similar slope for the fast part of the transient, while the slow part of the transient revealed an obvious difference between CLL-grown and FL-grown plants. CLL-grown plants showed a substantial slowdown in the slow phase of transient compared with FL-grown plants. Two additional parameters that can be derived from MR_820_ signals, V_ox_ and V_red_ (Figure 4B), represent the oxidation rate of PC and P700 and the re-reduction rate of PC^+^ and P700^+^, respectively. FL-grown plants showed a significantly higher V_ox_ value, while the V_red_ was significantly lower compared with CLL-grown plants.

### Rate of Electron Transport and Quantum Efficiencies of the Photosystem II

**FIGURE 5:**
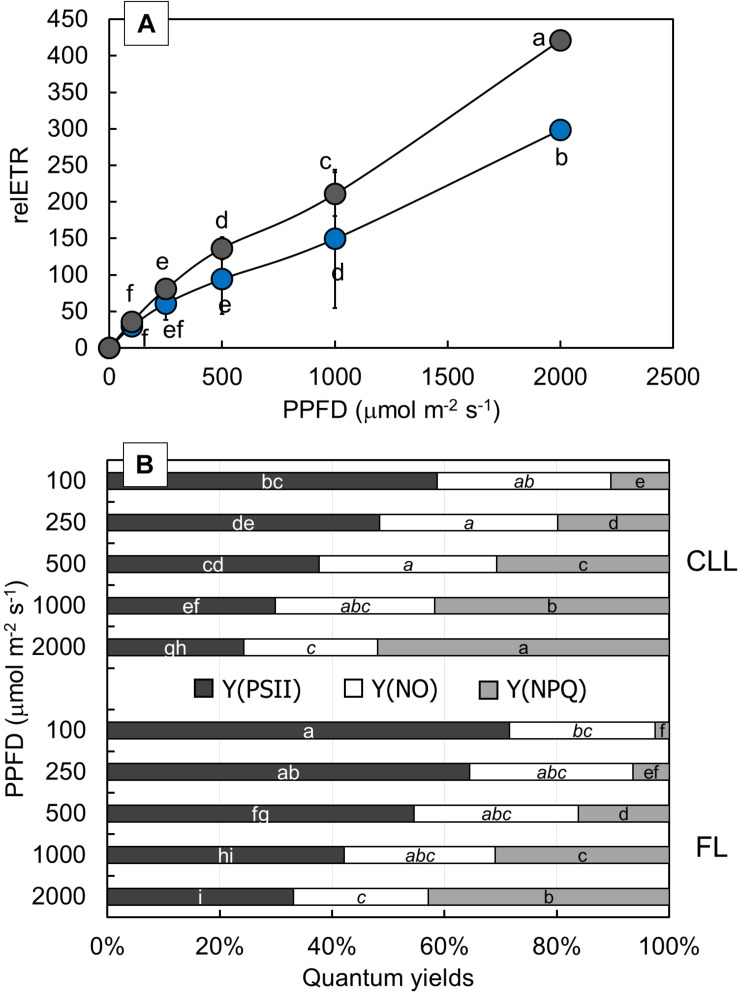
Changes of relative electron transport rate [relETR **(A)**] were measured at 100, 250, 500, 1,000, and 2,000 μmol/m^2^/s in the Japanese knotweed (*Reynoutria japonica* Houtt.) leaves exposed to constant low light (blue dots) and fluctuating light (gray dots). Complementary changes of quantum yields **(B)**: quantum efficiency of PSII photochemistry (Y(PSII), dark gray), quantum yield of non-regulated energy dissipated in PSII (Y(NO), white), and quantum yield for dissipation through downregulation in PSII (Y(NPQ), light gray) were represented for each applied light intensity for both investigated plant groups. Data are shown as means of six replicates (*n* = 6). Different letters represent significant difference at *p* ≤ 0.05 (using the ANOVA LSD test) between two investigated plant groups at each light intensity within each quantum yield [white letters for Y(PSII), letters in italic for Y(NO), and black letters for Y(NPQ)].

To determine the effect of light intensity on the PSII activity, relETR, Y(PSII), Y(NO), and Y(NPQ) were measured at different light intensities (Figure 5). FL-grown plants showed significantly higher relETR values (Figure 5A) at moderate (500 PPFD) and high light intensities (1,000 and 2,000 PPFD), while at lower light intensities (100 and 250 PPFD), there was no significant difference between CLL-grown and FL-grown plants. The effective photochemical quantum yield of PSII [Y(PSII)], the quantum yield of non-regulated energy dissipation [Y(NO)], and the quantum yield for dissipation by downregulation [Y(NPQ)] describe the energy distribution through PSII (Figure 5B). Both CLL-grown and FL-grown plants revealed a similar response of measured parameters. Nevertheless, there was a significant difference for Y(PSII) and Y(NPQ) parameters at all applied light intensities between both the plant groups. Except for the significantly lower Y(NO) measured at 2,000 PPFD compared with the lower light intensities in CLL-grown plants, Y(NO) showed that there was no significant change regardless of the applied light intensity between CLL-grown and FL-grown plants.

### Content of Photosynthetic Pigments

**FIGURE 6:**
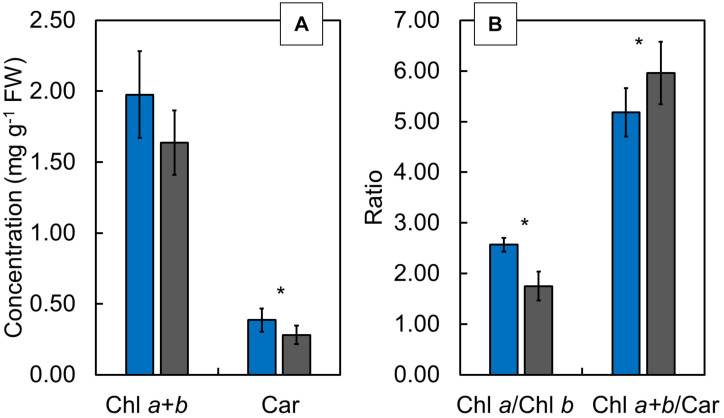
The Chl *a* + *b* (mg/g FW) and carotenoids (Car; mg/g FW) **(A)**, as well as Chl *a/b* and Chl *a* + *b*/Car ratios **(B)**, determined in the Japanese knotweed (*Reynoutria japonica* Houtt.) leaves exposed to constant low light (blue bars) and fluctuating light (gray bars). The values are represented as means ± SD. The asterisk (*) represents a significant difference at *p* ≤ 0.05 (using the Student’s *t*-test).

Although the Chl *a* + *b* showed no significant difference between CLL-grown and FL-grown plants (Figure 6A), the Car (Figure 6A) and the Chl *a*/*b* ratio (Figure 6B) showed significantly lower values, while the Chl *a* + *b*/Car ratio (Figure 6B) revealed significantly higher values in FL-grown plants compared with CLL-grown plants.

## Discussion

The Japanese knotweed plants grown in CLL and FL conditions exhibited a differential response of photosynthetic light-dependent reactions. Our results suggested that the knotweed plants grown in FL acclimated under such conditions by showing better overall photosynthetic reactions compared with plants grown in CLL, thus showing high acclimation potential. FL-grown knotweeds showed a protected integrity of thylakoid membranes, showing better grouping and connectivity between the reaction centers of PSII. This allows them for an efficient allocation of absorbed energy that can be efficiently utilized in primary photochemistry. A larger acceptor pool enables them to achieve an efficient electron transport due to higher amount of free-electron acceptors, as well as more efficient reduction rate at the PSI acceptor side. The fully functional OEC enables replacement of sufficient amount of electrons toward PSII to drive the functional photosynthetic reactions. As a result, FL-grown knotweeds revealed a functional electron transport all the way to PSI, as well as enhanced the photochemical activity of PSI at the acceptor side. In addition, the results after short-term light treatments suggested that FL-grown plants generate a cyclic electron flow to protect PSI by preventing overreduction of the PSI acceptor side.

When exposed to different growth conditions, the photosynthetic apparatus comprises various strategies for adaptation and/or protection at different levels of light conversion throughout the electron transport chain. The OJIP transient curves give us the perception of the status of the plant; therefore, its shape was shown to be a good indicator of the pool size of the electron carriers in the photosynthetic electron transport chain. Stressful conditions, therefore, cause the change in the intensity of the characteristic points of the OJIP curve. Subsequently, the intensity in the J, I, and P steps, and also in the intermediate L and K bands, changes (Strasser et al., 2000; Kalaji et al., 2018b). It was proposed that low light induces higher J step and, hence, higher V_J_ and also higher ψ_E__0_ due to the limited re-oxidation of Q_A_ (Strasser et al., 2007). Therefore, a smaller pool of plastoquinone (PQ) disables CLL-grown knotweeds to achieve an efficient electron transport since they had a lower amount of free-electron acceptors (Gao et al., 2014). Stress can induce higher I step due to the accumulation of a higher amount of reduced Q_A_ and PQ, which subsequently blocks the electron transport between Q_A_ and Q_B_ and further to PSI (Strasser et al., 2010; Kalaji et al., 2014). It was reported for the Norway spruce vegetative buds (Katanić et al., 2012), developing common fig leaves (Mlinarić et al., 2017), and radish plants exposed to sulfur deficiency (Samborska et al., 2019) that V_I_ increase is connected with reoxidation and turnover rate. Based on that, our results imply that Q_A_ of CLL-grown plants could be reduced, but not reoxidized as efficient as FL-grown plants.

The good grouping and connectivity between the reaction centers of PSII enable an efficient allocation of the absorbed energy to the primary acceptor Q_A_ (Yusuf et al., 2010). The occurrence of the K-band and the parameter F_v_/F_0_ reflects the activity of the OEC on the donor site of PSII (Kalaji et al., 2011). Therefore, the tolerance to various stress, such as salinity (Da̧browski et al., 2019) or drought (Oukarroum et al., 2007), is often connected with the appearance of a negative L-band. Likewise, a negative K-band is often associated with the plants that exhibit tolerance to stress such as heavy metals (Żurek et al., 2014; Begović et al., 2016), salinity (Pavlović et al., 2019), chilling (Krüger et al., 2014), and drought (Oukarroum et al., 2009; Begović et al., 2020), suggesting that functional OEC can replace a sufficient amount of electrons toward PSII to drive functional photosynthetic reactions. However, recent investigation on low-light-grown and high-light-grown *Phalenopsis* plants revealed a lower P_2__G_ in high-light-grown plants (Ceusters et al., 2019), suggesting the higher connectivity under the light limitation. Our results, however, suggested that FL-grown plants had closely connected thylakoids that are considered stable and not likely to undergo structural changes. The negative shape of the L-band is the reliable indicator of better grouping and connectivity between the reaction centers of PSII that enables an efficient allocation of the absorbed energy to the primary acceptor Q_A_ (Yusuf et al., 2010). Such closely connected thylakoids are considered stable and not likely to undergo structural changes. Therefore, the negative L-band and higher, although not significantly, P_2__G_, the overall grouping probability within the PSII antennae in FL-grown plants suggested better grouping and connectivity between the reaction centers of PSII in FL-grown plants, which is related with the preservation of integrity of thylakoid membranes in FL-grown plants. In addition, FL-grown plants carry out efficient photosynthetic reactions due to fully functional OEC that was able to replace necessary amount of electrons in the direction of PSII.

The most recent investigations also involved the calculations of H- and G-bands. The H-band is connected to the redox state of the Q_A_, and the negative amplitude is the result of the inhibited reoxidation of Q_A_^–^ (Kalaji et al., 2018a; Da̧browski et al., 2019). Negative amplitudes of G-band were associated with the adaptation mechanism in nutrient-deficient rapeseed plants that compensate the functionality by increasing the number of NADP^+^ molecules per active RC (Kalaji et al., 2018a). A similar response of FL-grown plants in our investigation suggests certain adaptation to the FL. Additionally, the maximal amplitude of W_OI_ ≥ 1 reveals the IP phase, where larger amplitudes suggest larger acceptors pool (Yusuf et al., 2010; Guo et al., 2020). Therefore, a higher W_OI_ amplitude in FL-grown plants indicated a bigger pool of the end electron acceptors at the PSI acceptor side compared with CLL-grown plants. Our results for FL-grown plants were consistent with those implicating more efficient reduction rate at the PSI acceptor side in FL-grown knotweeds compared with CLL-grown knotweeds.

Recently, it was suggested that a good connection of PSII would ensure an efficient utilization of absorbed light into the electron transport and the excitation energy from closed RCs will be transferred to open ones, but without connectivity, the excitation energy will be mainly dissipated. Hence, an increased connectivity is often associated with more efficient processing of light energy (Ceusters et al., 2019). In our investigation, considering connectivity, FL-grown plants showed a similar behavior pattern as HL-grown *Phalenopsis*, indicating that light limitation of CLL-grown plants diminished energy fluctuations through PSII. Such specific fluxes consider only active RCs that can reduce Q_A_ (Force et al., 2003; Yusuf et al., 2010). A significantly lower Chl *a/b* ratio in FL-grown plants suggests a higher acclimation potential due to the formation of smaller, but more efficient, photosynthetic units (Oguchi et al., 2003; Brestič et al., 2014). Furthermore, F_m_ and Chl *a* + *b* did not differ between FL-grown and CCL-grown plants. It was suggested that there was a strong correlation between F_m_ and Chl content and that changes in the chlorophyll content do not affect the antenna size but reflect its ability to acclimate to the light environment (Dinç et al., 2012). FL-grown knotweed plants generated an efficient mechanism to regulate the amount of excitation energy needed to reach the RC as acclimation to fluctuations in the light intensity. Moreover, the absorbed light energy in FL-grown knotweeds was efficiently utilized in the primary photochemistry and revealed functional electron transport all the way to PSI.

The PI_total_ was known to be the most sensitive parameter that describes the functional activity of PSII, PSI, and intersystem electron transport chain (Yusuf et al., 2010; Krüger et al., 2014; Da̧browski et al., 2016), and therefore, it allows the extensive analyses of the photosynthetic performance. It was suggested that a higher value of log φ_P__0_/(1-φ_P__0_) is associated with an efficient primary photochemistry due to the light reactions (Pereira et al., 2000; Kalaji et al., 2011). An increase of this log ψ_E__0_/(1-ψ_E__0_) suggests the improved ability of the photosynthetic system for the conversion of the excitation energy to electron transport beyond Q_A_^–^ in plants grown in the FL (van Heerden et al., 2007; Krüger et al., 2014). Based on that, our results imply that FL-grown plants revealed highly regulated photosynthetic processes between the light-dependent reactions and the reactions leading to CO_2_ assimilation compared with CCL-grown ones.

The modulated reflection at 820 nm represents the oxidation state of PC and P700 and re-reduction state of PC^+^ and P700^+^ and depends on the available pool of electron acceptors on the acceptor side of PSI (Strasser et al., 2010; Guo et al., 2020). The faster oxidation of PC and P700 reflects the enhanced photochemical activity of PSI at the acceptor side (Gao et al., 2014; Salvatori et al., 2015). It was suggested that a higher PSI activity could be an adaptive mechanism for minimizing the photooxidative damage by regulating the distribution of excitation energy between PSII and PSI (Zhang et al., 2016). It was reported recently that FL primarily damages PSI in the wild-type *Arabidopsis* plants. In that case, the generation of the cyclic electron flow around PSI could play an important role in the photoprotection of the PSI donor side (Yamamoto and Shikanai, 2019). Therefore, based on the above-mentioned studies, our results suggested that in FL-grown plants, there were too few electrons transferred to PSI, which were not able to completely reduce P700^+^ and PC^+^ compared with CLL-grown knotweeds.

The photosynthetic efficiency is known to decrease under high irradiation, and at the same time, heat dissipation and the relative electron transport increase (Bajkán et al., 2012; Sperdouli and Moustakas, 2012; Brestič et al., 2014; Huang et al., 2018). Several components can be involved in the increase of non-photochemical quenching, and most usually, its increase is associated with the dissipation of the active energy *via* the carotenoids (Demmig-Adams and Adams, 1996; Brestič et al., 2014). CLL-grown plants dissipated greater amount of energy by downregulation, implying an effective mechanism to cope with the photoinhibitory conditions. Such an increase could be the case in our investigation since the higher Car, as well as higher Chl *a* + *b* to Car ratio, was observed in CLL-grown plants. Recently, it was suggested that such a mechanism could be directly associated with the P700 redox status (Brestič et al., 2014; Zhang et al., 2016). The short-term exposure of FL-grown knotweeds could cause accumulation of electrons in PSI, resulting in oxidative damage. To prevent overreduction of the PSI acceptor side, cyclic electron flow limits the production of reactive oxygen species, thus protecting the acceptor side of PSI (Salvatori et al., 2015). In such case, FL-grown plants can generate a cyclic electron flow to protect PSI (Yamamoto and Shikanai, 2019).

The parallel measurements of the photosynthetic parameters used several non-destructive methods, and the determination of photosynthetic pigments showed that CLL led to a lower functionality of the light-driven photosynthetic reaction in the Japanese knotweed compared with plants grown in FL. The growth in FL, however, induced fully efficient PSII and PSI, reaction centers, and intersystem electron transport. To our knowledge, obtained results in this investigation revealed the most detailed insight into the light-driven reactions in the invasive Japanese knotweed and the adaptations of the photosynthetic apparatus to the FL conditions. Recent study on the growth dynamics of this invasive species based on the light availability showed that the Japanese knotweed grew faster and explored larger area when cultivated in full sunlight (Martin et al., 2020). They found that plants grown in full sunlight had higher vigor compared with those grown in shaded area. This corresponds to our findings that CLL-grown plants had a poor photosynthetic performance compared with FL-grown plants, which could be one of the key roles of its invasive success.

## Conclusion

It can be concluded that Japanese knotweed plants grown in FL built distinct adaptations to the changing light conditions compared with the plants grown in CLL. Our results revealed that FL exhibited more efficient photosynthetic reactions compared with the plants grown in CLL due to the better grouping and connectivity between the PSII units compared with CLL-grown plants. The fully functional OEC in FL-grown plants was able to replace a sufficient amount of electrons toward PSII to drive functional photochemical reactions. The formation of smaller photosynthetic units in FL-grown plants caused a lower absorption and trapping but a more efficient conversion of excitation energy to the electron transport beyond the primary electron acceptor Q_A_. An efficient reduction and reoxidation of Q_A_ in FL-grown plants ensured rather undisturbed electron transport all the way to PSI, while the larger PQ pool enabled them to achieve an efficient electron transport due to a higher amount of free-electron acceptors. Due to the larger acceptor pool at the PSI acceptor side, FL-grown plants were more capable of reduction of their end acceptor than CLL-grown ones. The enhanced photochemical activity of PSI in FL-grown plants suggested the formation of a successful adaptive mechanism for minimizing the photooxidative damage by regulating the distribution of the excitation energy between PSII and PSI. In contrast, CLL-grown plants accumulated a higher amount of reduced Q_A_ and PQ that could be reduced but not reoxidized as efficient as in FL-grown plants. That subsequently blocked the electron transport between Q_A_ and Q_B_ and further to PSI. However, FL-grown knotweeds exhibited faster oxidation at the PSI side, which could be the result of generating the cyclic electron flow around PSI. Despite the better effective quantum yield of PSII and the linear electron transport observed in FL-grown plants, an exposure to the short-term high light intensity increased Y(NPQ), the yield induced by the downregulatory processes, suggesting that the generation of the cyclic electron flow around PSI was due to the functional adaptation of the FL-grown plants to protect PSI from photoinhibition.

## Author Contributions

SM proposed a conceptual framework, supervised the research, analyzed the data, and wrote the manuscript. LB performed measurements and statistical analysis, reviewed, and edited the manuscript. NT and AP performed the measurements. VC reviewed the manuscript. All authors contributed to the manuscript revision, read, and approved the submitted version.

## Conflict of Interest

The authors declare that the research was conducted in the absence of any commercial or financial relationships that could be construed as a potential conflict of interest.

## Publisher’s Note

All claims expressed in this article are solely those of the authors and do not necessarily represent those of their affiliated organizations, or those of the publisher, the editors and the reviewers. Any product that may be evaluated in this article, or claim that may be made by its manufacturer, is not guaranteed or endorsed by the publisher.

## References

[B1] Armbruster U. Galvis V. C. Kunz H.-H. Strand D. D. (2017). The regulation of the chloroplast proton motive force plays a key role for photosynthesis in fluctuating light. *Curr. Opin. Plant Biol* 37 56–62. 10.1016/j.pbi.2017.03.012 28426975

[B2] Bajkán S. Várkonyi Z. Lehoczki E. (2012). Comparative study on energy partitioning in photosystem II of two Arabidopsis thaliana mutants with reduced non-photochemical quenching capacity. *Acta Physiol. Plant* 34 1027–1034. 10.1007/s11738-011-0899-1

[B3] Bajwa A. A. Chauhan B. S. Farooq M. Shabbir A. Adkins S. W. (2016). What do we really know about alien plant invasion? A review of the invasion mechanism of one of the world’s worst weeds. *Planta* 244 39–57. 10.1007/s00425-016-2510-x 27056056

[B4] Barney J. N. Tharayil N. DiTommaso A. Bhowmik P. C. (2006). The biology of invasive alien plants in Canada. 5. Polygonum cuspidatum Sieb. & Zucc.[= Fallopia japonica (Houtt.) Ronse Decr.]. *Can. J. Plant Sci.* 86 887–906. 10.4141/P05-170

[B5] Beerling D. J. Bailey J. P. Conolly A. P. (1994). *Fallopia japonica* (Houtt.) ronse decraene. *J. Ecol.* 82 959–979. 10.2307/2261459

[B6] Begović L. Galić V. Abičić I. Lončarić Z. Lalić A. Mlinarić S. (2020). Implications of intra-seasonal climate variations on chlorophyll a fluorescence and biomass in winter barley breeding program. *Photosynthetica* 58 995–1008. 10.32615/ps.2020.053

[B7] Begović L. Mlinarić S. Antunović Dunić J. Katanić Z. Lončarić Z. Lepeduš H. (2016). Response of *Lemna minor* L. to short-term cobalt exposure: the effect on photosynthetic electron transport chain and induction of oxidative damage. *Aquat. Toxicol.* 175 117–126. 10.1016/j.aquatox.2016.03.009 27015565

[B8] Bilger W. Björkman O. (1990). Role of the xanthophyll cycle in photoprotection elucidated by measurements of light-induced absorbance changes, fluorescence and photosynthesis in leaves of Hedera canariensis. *Photosynth. Res.* 25 173–185. 10.1007/BF00033159 24420348

[B9] Boršić I. Milović M. Dujmović I. Bogdanović S. Cigić P. Rešetnik I. (2008). Preliminary check-list of invasive alien plant species (IAS) in Croatia. *Nat. Croat.* 17 55–71.

[B10] Brestič M. Živčak M. Olsovska K. Shao H.-B. Kalaji H. M. Allakhverdiev S. I. (2014). Reduced glutamine synthetase activity plays a role in control of photosynthetic responses to high light in barley leaves. *Plant Physiol. Biochem.* 81 74–83. 10.1016/j.plaphy.2014.01.002 24491798

[B11] Bussotti F. Pollastrini M. (2017). Observing climate change impacts on European forests: what works and what does not in ongoing long-term monitoring networks. *Front. Plant Sci.* 8:629. 10.3389/fpls.2017.00629 28487718 PMC5404609

[B12] CABI (2019). *Fallopia Japonica (Japanese Knotweed)* [Online]. Available online at: https://www.cabi.org/isc/datasheet/23875 (accessed September 23, 2020).

[B13] Ceusters N. Valcke R. Frans M. Claes J. E. Van den Ende W. Ceusters J. (2019). Performance index and PSII connectivity under drought and contrasting light regimes in the CAM orchid phalaenopsis. *Front. Plant Sci.* 10:1012. 10.3389/fpls.2019.01012 31447875 PMC6691161

[B14] Da̧browski P. Baczewska A. H. Pawluśkiewicz B. Paunov M. Alexantrov V. Goltsev V. (2016). Prompt chlorophyll a fluorescence as a rapid tool for diagnostic changes in PSII structure inhibited by salt stress in *Perennial ryegrass*. *J. Photochem. Photobiol. B Biol.* 157 22–31. 10.1016/j.jphotobiol.2016.02.001 26878219

[B15] Da̧browski P. Baczewska-Da̧browska A. H. Kalaji H. M. Goltsev V. Paunov M. Rapacz M. (2019). Exploration of chlorophyll a fluorescence and plant gas exchange parameters as indicators of drought tolerance in perennial ryegrass. *Sensors* 19:2736. 10.3390/s19122736 31216685 PMC6631610

[B16] Demmig-Adams B. Adams W. W. (1996). The role of xanthophyll cycle carotenoids in the protection of photosynthesis. *Trends Plant Sci.* 1 21–26. 10.1016/S1360-1385(96)80019-7

[B17] Desotgiu R. Cascio C. Pollastrini M. Gerosa G. Marzuoli R. Bussotti F. (2012). Short and long term photosynthetic adjustments in sun and shade leaves of *Fagus sylvatica* L., investigated by fluorescence transient (FT) analysis. *Plant Biosyst.* 146 206–216. 10.1080/11263504.2012.705350

[B18] Dinç E. Ceppi M. G. Tóth S. Z. Bottka S. Schansker G. (2012). The chl a fluorescence intensity is remarkably insensitive to changes in the chlorophyll content of the leaf as long as the chl a/b ratio remains unaffected. *Biochim. Biophys. Acta Biomembr.* 1817 770–779. 10.1016/j.bbabio.2012.02.003 22342617

[B19] Dommanget F. Evette A. Breton V. Daumergue N. Forestier O. Poupart P. (2019). Fast-growing willows significantly reduce invasive knotweed spread. *J. Environ. Manage.* 231 1–9. 10.1016/j.jenvman.2018.10.004 30326333

[B20] Dommanget F. Spiegelberger T. Cavaillé P. Evette A. (2013). Light availability prevails over soil fertility and structure in the performance of Asian knotweeds on riverbanks: new management perspectives. *Environ. Manage.* 52 1453–1462. 10.1007/s00267-013-0160-3 24065383

[B21] FCD (2020). *Flora Croatica Database.* Available online at: https://hirc.botanic.hr/fcd/DetaljiFrame.aspx?IdVrste=8449&taxon=Reynoutria+japonica+Houtt (accessed September 23, 2020).

[B22] Force L. Critchley C. van Rensen J. J. (2003). New fluorescence parameters for monitoring photosynthesis in plants. *Photosynth. Res.* 78 17–33. 10.1023/A:1026012116709 16245061

[B23] Gao J. Li P. Ma F. Goltsev V. (2014). Photosynthetic performance during leaf expansion in *Malus micromalus* probed by chlorophyll a fluorescence and modulated 820 nm reflection. *J. Photochem. Photobiol. B Biol.* 137 144–150. 10.1016/j.jphotobiol.2013.12.005 24373888

[B24] Genty B. Briantais J.-M. Baker N. R. (1989). The relationship between the quantum yield of photosynthetic electron transport and quenching of chlorophyll fluorescence. *Biochim. Biophys. Acta Gen. Subj.* 990 87–92. 10.1016/S0304-4165(89)80016-9

[B25] Goltsev V. Kalaji H. Paunov M. Ba̧ba W. Horaczek T. Mojski J. (2016). Variable chlorophyll fluorescence and its use for assessing physiological condition of plant photosynthetic apparatus. *Russ. J. Plant Physiol.* 63 869–893. 10.1134/S1021443716050058

[B26] Guo Y. Lu Y. Goltsev V. Strasser R. J. Kalaji H. M. Wang H. (2020). Comparative effect of tenuazonic acid, diuron, bentazone, dibromothymoquinone and methyl viologen on the kinetics of Chl a fluorescence rise OJIP and the MR820 signal. *Plant Physiol. Biochem.* 156 39–48. 10.1016/j.plaphy.2020.08.044 32906020

[B27] Huang W. Yang Y.-J. Zhang S.-B. Liu T. (2018). Cyclic electron flow around photosystem I promotes ATP synthesis possibly helping the rapid repair of photodamaged photosystem II at low light. *Front. Plant Sci.* 9:239. 10.3389/fpls.2018.00239 29535751 PMC5834426

[B28] Kaiser E. Morales A. Harbinson J. (2018). Fluctuating light takes crop photosynthesis on a rollercoaster ride. *Plant Physiol.* 176 977–989. 10.1104/pp.17.01250 29046421 PMC5813579

[B29] Kalaji H. M. Ba̧ba W. Gediga K. Goltsev V. Samborska I. A. Cetner M. D. (2018a). Chlorophyll fluorescence as a tool for nutrient status identification in rapeseed plants. *Photosynth. Res.* 136 329–343. 10.1007/s11120-017-0467-7 29185137 PMC5937862

[B30] Kalaji H. M. Bosa K. Kościelniak J. Żuk-Gołaszewska K. (2011). Effects of salt stress on photosystem II efficiency and CO2 assimilation of two Syrian barley landraces. *Environ. Exp. Bot.* 73 64–72. 10.1016/j.envexpbot.2010.10.009

[B31] Kalaji H. M. Oukarroum A. Alexandrov V. Kouzmanova M. Brestic M. Zivcak M. (2014). Identification of nutrient deficiency in maize and tomato plants by in vivo chlorophyll a fluorescence measurements. *Plant Physiol. Biochem.* 81 16–25. 10.1016/j.plaphy.2014.03.029 24811616

[B32] Kalaji H. M. Rastogi A. Živčák M. Brestic M. Daszkowska-Golec A. Sitko K. (2018b). Prompt chlorophyll fluorescence as a tool for crop phenotyping: an example of barley landraces exposed to various abiotic stress factors. *Photosynthetica* 56 953–961. 10.1007/s11099-018-0766-z

[B33] Katanić Z. Atić L. Ferhatović D. Cesar V. Lepeduš H. (2012). PSII photoscemistry in vegetative buds and needles of Norway spruce (*Picea abies* L. Karst.) probed by OJIP chlorophyll a fluorescence measurement. *Acta Biol. Hung.* 63 218–230. 10.1556/abiol.63.2012.2.5 22695521

[B34] Keren N. Berg A. Van Kan P. J. Levanon H. Ohad I. (1997). Mechanism of photosystem II photoinactivation and D1 protein degradation at low light: the role of back electron flow. *Proc. Natl. Acad. Sci. U.S.A.* 94 1579–1584. 10.1073/pnas.94.4.1579 11038602 PMC19834

[B35] Kouřil R. Wientjes E. Bultema J. B. Croce R. Boekema E. J. (2013). High-light vs. low-light: effect of light acclimation on photosystem II composition and organization in *Arabidopsis thaliana*. *Biochim. Biophys Acta Biomembr.* 1827 411–419. 10.1016/j.bbabio.2012.12.003 23274453

[B36] Kramer D. M. Johnson G. Kiirats O. Edwards G. E. (2004). New fluorescence parameters for the determination of QA redox state and excitation energy fluxes. *Photosynth. Res.* 79:209. 10.1023/B:PRES.0000015391.99477.0d 16228395

[B37] Krüger G. H. J. De Villiers M. F. Strauss A. J. de Beer M. van Heerden P. D. R. Maldonado R. (2014). Inhibition of photosystem II activities in soybean (*Glycine max*) genotypes differing in chilling sensitivity. *S. Afr. J. Bot.* 95 85–96. 10.1016/j.sajb.2014.07.010

[B38] Li Q. Xiao H. (2012). The interactions of soil properties and biochemical factors with plant allelopathy. *Ecol. Environ. Sci.* 21 2031–2036.

[B39] Lichtenthaler H. K. (1987). Chlorophylls and carotenoids: pigments of photosynthetic biomembranes. *Methods Enzymol.* 148 350–382.

[B40] Lichtenthaler H. K. Ač A. Marek M. V. Kalina J. Urban O. (2007). Differences in pigment composition, photosynthetic rates and chlorophyll fluorescence images of sun and shade leaves of four tree species. *Plant Physiol. Biochem.* 45 577–588. 10.1016/j.plaphy.2007.04.006 17587589

[B41] Lichtenthaler H. K. Burkart S. (1999). Photosynthesis and high light stress. *Bulgarian J. Plant Physiol.* 25 3–16.

[B42] Martin F.-M. Dommanget F. Lavallée F. Evette A. (2020). Clonal growth strategies of Reynoutria japonica in response to light, shade, and mowing, and perspectives for management. *NeoBiota* 56 89–110.

[B43] Mathur S. Allakhverdiev S. I. Jajoo A. (2011). Analysis of high temperature stress on the dynamics of antenna size and reducing side heterogeneity of Photosystem II in wheat leaves (*Triticum aestivum*). *Biochim. Biophys. Acta Biomembr.* 1807 22–29. 10.1016/j.bbabio.2010.09.001 20840840

[B44] Matthews J. S. Vialet-Chabrand S. Lawson T. (2018). Acclimation to fluctuating light impacts the rapidity of response and diurnal rhythm of stomatal conductance. *Plant Physiol.* 176 1939–1951. 10.1104/pp.17.01809 29371250 PMC5841698

[B45] Mlinarić S. Antunović Dunić J. Skendrović Babojelić M. Cesar V. Lepeduš H. (2017). Differential accumulation of photosynthetic proteins regulates diurnal photochemical adjustments of PSII in common fig (*Ficus carica* L.) leaves. *J. Plant Physiol.* 209 1–10. 10.1016/j.jplph.2016.12.002 27987432

[B46] Oguchi R. Hikosaka K. Hirose T. (2003). Does the photosynthetic light-acclimation need change in leaf anatomy? *Plant Cell Environ.* 26 505–512. 10.1046/j.1365-3040.2003.00981.x

[B47] Oukarroum A. El Madidi S. Schansker G. Strasser R. J. (2007). Probing the responses of barley cultivars (*Hordeum vulgare* L.) by chlorophyll a fluorescence OLKJIP under drought stress and re-watering. *Environ. Exp. Bot.* 60 438–446. 10.1016/j.envexpbot.2007.01.002

[B48] Oukarroum A. Goltsev V. Strasser R. J. (2013). Temperature effects on pea plants probed by simultaneous measurements of the kinetics of prompt fluorescence, delayed fluorescence and modulated 820 nm reflection. *PLoS One* 8:e59433. 10.1371/journal.pone.0059433 23527194 PMC3602342

[B49] Oukarroum A. Schansker G. Strasser R. J. (2009). Drought stress effects on photosystem I content and photosystem II thermotolerance analyzed using Chl a fluorescence kinetics in barley varieties differing in their drought tolerance. *Physiol. Plant.* 137 188–199. 10.1111/j.1399-3054.2009.01273.x 19719481

[B50] Pavlović I. Mlinarić S. Tarkowská D. Oklestkova J. Novak O. Lepeduš H. (2019). Early Brassica crops responses to salinity stress: a comparative analysis between Chinese cabbage, white cabbage and kale. *Front. Plant Sci.* 10:450. 10.3389/fpls.2019.00450 31031786 PMC6470637

[B51] Pereira W. E. de Siqueira D. L. Martínez C. A. Puiatti M. (2000). Gas exchange and chlorophyll fluorescence in four citrus rootstocks under aluminium stress. *J. Plant Physiol.* 157 513–520. 10.1016/S0176-1617(00)80106-6

[B52] Pollastrini M. Nogales A. G. Benavides R. Bonal D. Finer L. Fotelli M. (2017). Tree diversity affects chlorophyll a fluorescence and other leaf traits of tree species in a boreal forest. *Tree Physiol.* 37 199–208. 10.1093/treephys/tpw132 28100710

[B53] Price E. A. Gamble R. Williams G. G. Marshall C. (2002). “Seasonal patterns of partitioning and remobilization of 14 C in the invasive rhizomatous perennial Japanese knotweed (*Fallopia japonica* (Houtt.) Ronse Decraene),” in *Ecology and Evolutionary Biology of Clonal Plants*, eds Stuefer J. F. Erschbamer B. Huber H. Suzuki J. I. (Dordrecht: Springer), 125–140.

[B54] Salvatori E. Fusaro L. Gottardini E. Pollastrini M. Goltsev V. Strasser R. J. (2014). Plant stress analysis: application of prompt, delayed chlorophyll fluorescence and 820 nm modulated reflectance. Insights from independent experiments. *Plant Physiol. Biochem.* 85 105–113. 10.1016/j.plaphy.2014.11.002 25463266

[B55] Salvatori E. Fusaro L. Strasser R. J. Bussotti F. Manes F. (2015). Effects of acute O3 stress on PSII and PSI photochemistry of sensitive and resistant snap bean genotypes (*Phaseolus vulgaris* L.), probed by prompt chlorophyll “a” fluorescence and 820 nm modulated reflectance. *Plant Physiol. Biochem.* 97 368–377. 10.1016/j.plaphy.2015.10.027 26535554

[B56] Samborska I. A. Kalaji H. M. Sieczko L. Borucki W. Mazur R. Kouzmanova M. (2019). Can just one-second measurement of chlorophyll a fluorescence be used to predict sulphur deficiency in radish (*Raphanus sativus* L. sativus) plants?. *Curr. Plant Biol.* 19:100096. 10.1016/j.cpb.2018.12.002

[B57] Schneider T. Bolger A. Zeier J. Preiskowski S. Benes V. Trenkamp S. (2019). Fluctuating light interacts with time of day and leaf development stage to reprogram gene expression. *Plant Physiol.* 179 1632–1657. 10.1104/pp.18.01443 30718349 PMC6446761

[B58] Sperdouli I. Moustakas M. (2012). Differential response of photosystem II photochemistry in young and mature leaves of *Arabidopsis thaliana* to the onset of drought stress. *Acta Physiol. Plant.* 34 1267–1276. 10.1007/s11738-011-0920-8

[B59] Spiering D. J. (2011). Effectiveness of two alternative herbicides compared to a conventional chemical herbicide for control of Japanese knotweed (*Polygonum cuspidatum*). *Bull. Buffalo Soc. Nat. Sci.* 40 49–58.

[B60] Strasser R. J. Srivastava A. Tsimilli-Michael M. (2000). “The fluorescence transient as a tool to characterize and screen photosynthetic samples,” in *Probing Photosynthesis: Mechanism, Regulation & Adaptation*, 1st Edn, eds Yunus M. Pathre U. Mohanty P. (New York, NY: CRC), 445–483.

[B61] Strasser R. J. Tsimilli-Michael M. Dangre D. Rai M. (2007). “Biophysical phenomics reveals functional building blocks of plants systems biology: a case study for the evaluation of the impact of mycorrhization with Piriformospora indica,” in *Advanced Techniques in Soil Microbiology*, eds Varma A. Oelmüller R. (Berlin: Springer Verlag), 319–341.

[B62] Strasser R. J. Tsimilli-Michael M. Qiang S. Goltsev V. (2010). Simultaneous in vivo recording of prompt and delayed fluorescence and 820-nm reflection changes during drying and after rehydration of the resurrection plant Haberlea rhodopensis. *Biochim. Biophys. Acta Bioenerg.* 1797 1313–1326. 10.1016/j.bbabio.2010.03.008 20226756

[B63] Strasser R. J. Tsimilli-Michael M. Srivastava A. (2004). “Analysis of the chlorophyll a fluorescence transient,” in *Chlorophyll a Fluorescence: A Signature of Photosynthesis*, eds Papageorgiou G. C. Govinjee (Dordrecht: Springer), 321–362.

[B64] Tomar R. Sharma A. Jajoo A. (2015). Assessment of phytotoxicity of anthracene in soybean (Glycine max) with a quick method of chlorophyll fluorescence. *Plant Biol.* 17 870–876. 10.1111/plb.12302 25565351

[B65] van Heerden P. D. R. Swanepoel J. W. Kruger G. H. J. (2007). Modulation of photosynthesis by drought in two desert scrub species exhibiting C3-mode CO2 assimilation. *Environ. Exp. Bot.* 61 124–136. 10.1016/j.envexpbot.2007.05.005

[B66] Yamamoto H. Shikanai T. (2019). PGR5-dependent cyclic electron flow protects photosystem I under fluctuating light at donor and acceptor sides. *Plant Physiol.* 179 588–600. 10.1104/pp.18.01343 30464024 PMC6426425

[B67] Yamori W. Kusumi K. Iba K. Terashima I. (2020). Increased stomatal conductance induces rapid changes to photosynthetic rate in response to naturally fluctuating light conditions in rice. *Plant Cell Environ.* 43 1230–1240. 10.1111/pce.13725 31990076

[B68] Yin Z.-H. Johnson G. N. (2000). Photosynthetic acclimation of higher plants to growth in fluctuating light environments. *Photosynth. Res.* 63 97–107. 10.1023/A:1006303611365 16252168

[B69] Yusuf M. A. Kumar D. Rajwanshi R. Strasser R. J. Tsimilli-Michael M. Govindjee (2010). Overexpression of γ-tocopherol methyl transferase gene in transgenic *Brassica juncea* plants alleviates abiotic stress: physiological and chlorophyll a fluorescence measurements. *Biochim. Biophys. Acta* 1797 1428–1438. 10.1016/j.bbabio.2010.02.002 20144585

[B70] Zhang D. Zhang Q. S. Yang X. Q. Sheng Z. T. Nan G. N. (2016). The alternation between PSII and PSI in ivy (*Hedera nepalensis*) demonstrated by in vivo chlorophyll a fluorescence and modulated 820 nm reflection. *Plant Physiol. Biochem.* 108 499–506. 10.1016/j.plaphy.2016.08.018 27592174

[B71] Živčak M. Brestič M. Kalaji H. M. (2014). Photosynthetic responses of sun-and shade-grown barley leaves to high light: is the lower PSII connectivity in shade leaves associated with protection against excess of light? *Photosynth. Res.* 119 339–354. 10.1007/s11120-014-9969-8 24445618 PMC3923118

[B72] Żurek G. Rybka K. Pogrzeba M. Krzyżak J. Prokopiuk K. (2014). Chlorophyll a fluorescence in evaluation of the effect of heavy metal soil contamination on *Perennial grasses*. *PLoS One* 9:e91475. 10.1371/journal.pone.0091475 24633293 PMC3954697

